# Evaluation of Over-the-Counter Cutaneous Lidocaine Cream for Temporary Deafferentation in Upper Limb Rehabilitation: A Cross-Sectional Study

**DOI:** 10.7759/cureus.94797

**Published:** 2025-10-17

**Authors:** Kelsey Baker, Daniel Salinas, Maria Lozano Bonilla, Jared Hensley, Hunter Butler, Monica Lozano Garcia, Chelsea Erazo, Ashley Tijerina, Victoria Cuello, Bharathi Gadad

**Affiliations:** 1 Neuroscience, The University of Texas Rio Grande Valley, Edinburg, USA; 2 Neurology, The University of Texas Rio Grande Valley School of Medicine, Harlingen, USA; 3 Medicine, The University of Texas Rio Grande Valley School of Medicine, Edinburg, USA; 4 Dermatology, The University of Texas Rio Grande Valley School of Medicine, Edinburg, USA; 5 Institute of Neuroscience, The University of Texas Rio Grande Valley, Harlingen, USA; 6 Medicine, The University of Texas Rio Grande Valley School of Medicine, Harlingen, USA; 7 Psychiatry and Behavioral Sciences, The University of Texas Rio Grande Valley School of Medicine, Edinburg, USA

**Keywords:** anesthesia, lidocaine, neurorehabilitation, optimization, rehabilitation, temporary deafferentation

## Abstract

Introduction: Topical or cutaneous methods of temporary deafferentation (TD) have shown potential as a tool to augment the effects of rehabilitative approaches for neurological conditions. TD has been suggested to achieve such effects due to its ability to suppress activity from afferent input that may be inhibitory to rehabilitative processes. However, most approaches to date have utilized prescription-grade anesthetic agents, which may be difficult to translate to routine outpatient rehabilitation or in remote settings. Here, we sought to evaluate whether TD could be achieved with readily available over-the-counter (OTC) 5% lidocaine topical anesthetic. We targeted the biceps brachii for TD, since previous work has suggested excessive afferent input from this muscle may limit triceps recovery following neurological injury.

Methods: Eighteen volunteers without any history of neurological disorders participated in a single-session, cross-sectional study design. TD was applied to the biceps brachii using 5% lidocaine cream (Ebanel). We assessed the loss of normal, diminished light touch, diminished protective sensation, and loss of protective sensation during TD using von Frey hair filaments (VFHT) for up to 75 minutes. Demographic factors (e.g., sex, age, fat percentage, and arm circumference) were evaluated as confounds for the level of achieved TD.

Results: Overall, TD was achieved after a minimum of 60 minutes following the application of OTC lidocaine, with normal and light touch showing the most significant loss in sensation. Age and sex were found to most significantly affect the level of achieved TD. We also found that the 4.56 weighted VFHT could be used to reliably determine if TD was achieved in 60 minutes.

Conclusions: Our results suggest that the use of OTC lidocaine can be used to achieve TD in as little as 60 minutes. While demographic factors were found to influence the level of TD, this approach offers a practical and economical solution for studies seeking to utilize TD outside of traditional healthcare or research facilities.

## Introduction

Temporary deafferentation (TD) has been used as a tool to understand injury-induced cortical reorganization in animal and clinical studies for over 50 years [1-4]. TD employs temporary anesthesia to suppress or lower the afferent input from a region, dermatome, or innervated muscle. In general, it is suggested that by suppressing or lowering such afferent input using TD, neural pathways that may be inhibited by dominant afferent input become unmasked. Specifically, Bjorkman et al. have demonstrated in a series of studies that cutaneous TD results in rapid cortical and subcortical reorganization [5]. Following application of TD to the forearm, functional magnetic resonance imaging (fMRI) showed that the contralateral primary somatosensory hand area was expanded cranially, medially, and posteriorly.

Several studies have used TD methodology to help define pathophysiological changes after injury in animal and clinical studies [5-13]. For example, Björkman and colleagues (2004) found that TD applied to the right hand resulted in increased activation in the right primary motor cortex, resulting in improved behavioral outcomes (e.g., tactility) and grip strength [5]. Similarly, Petoe and colleagues (2013) found that topical anesthesia on the forearm could improve sensory and motor function of the ipsilateral targeted hand [12] - a finding that has also been shown in populations of stroke survivors [14]. Mechanistic studies by Sehle and colleagues (2016) found that the application of unilateral upper limb TD modestly lengthened the cortical silent period (CSP) of the targeted side of the body, with no effect seen on the non-anesthetized side [15]. Similarly, Werhahn et al. found that TD resulted in changes in the processing of sensory information in the contralateral hemisphere to the TD-targeted muscle [7].

Neurorehabilitation studies have sought to capitalize on the mechanisms of TD to improve outcomes of traditional rehabilitation approaches [5,11,13,16-19]. In the application of nerve repair, Rosén et al. observed that cutaneous anesthesia on the forearm combined with sensory re-education in subjects who had ulnar or median nerve repair significantly enhanced sensory recovery in the hands of patients with nerve injury compared to placebo [13]. In stroke survivors, a combination of TD with constraint-induced movement therapy for a single day led to significantly improved outcomes for hand sensation and motor function [20]. Effects of multiple days of TD combined with practice-based movements have also been shown to significantly improve activities of daily living in chronic stroke survivors [21].

Methods to induce TD in neurorehabilitation studies have varied from non-invasive to invasive. Some commonly used methods include local ischemic nerve blocks in the form of tourniquets [6-8] and local anesthetic nerve blocks using injection [9,10] or topical cream [5,11-13]. One method that has gained more popularity in neurorehabilitation is the use of topical creams. The rationale for increased investigation of TD with topical cream may be linked to its minimal side effects from other methods (e.g., pain, practical difficulties, and inaccessibility) or the ease of application. Regardless, topically applied TD has shown promise in restoring sensory and motor losses with neurorehabilitation. For example, Petoe et al. found that topical forearm cutaneous anesthesia improved spatial acuity at the fingertips in healthy participants [12], a finding that has also been confirmed in stroke populations [22]. Additional studies in phantom limb pain and stroke have also shown the potential of topical creams to improve rehabilitation strategies [23-27].

Here, we sought to expand the translation of topically applied TD in rehabilitation settings and evaluate whether over-the-counter (OTC) anesthetic cream could be used to achieve TD. To date, topical TD approaches have commonly used a 5% eutectic mixture of local anesthetics (EMLA), with 2.5% lidocaine and 2.5% prilocaine. EMLA approaches require a prescription for dispensing of the cream [5,10-13,15,20,28]. Further, established topical TD protocols with EMLA have varied in the amount of cream used (1 to 20 grams) and duration (30 to 90 minutes). In addition, given the growing use of decentralized clinical trials and telemedicine [29,30], we sought to find a method that may be more feasible to implement in future studies. Our study sought to answer three key questions: (1) How much time is required to achieve maximum sensation loss with OTC lidocaine cream? (2) How do baseline characteristics influence the level of sensation loss? (3) Can the level of TD be ascertained using a simple one-step method? We hypothesized that OTC lidocaine cream could result in a significant decrease in sensory thresholds.

## Materials and methods

Study design and participants

**Figure 1:**
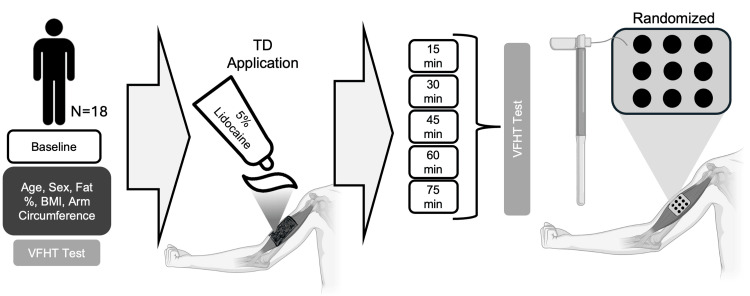
Study design. After enrollment, baseline characteristics (age, sex, fat percentage, body mass index, arm circumference, and baseline sensitivity with von Frey hair filaments (VFHT)) were assessed for all participants. Temporary deafferentation (TD) using over-the-counter (OTC) topical lidocaine cream was applied to the right biceps brachii and covered with Tegaderm. Every 15 minutes, for up to 75 minutes, sensitivity was assessed with 12 varying weights of VFHTs. Each filament was assessed in nine locations, in a random order, on the right biceps.

We conducted a single-session study to evaluate the use of OTC topical lidocaine for application in TD (Figure 1). The study took place at a clinical research laboratory located within a clinical outpatient facility. Participants were recruited from January 2020 to December 2023. Eighteen healthy volunteers without any history of upper limb injury and neurological disorders participated in this study. Sample size was estimated from previous work on topical TD in healthy controls [31]. Participants were eligible for inclusion if they were at least 18 years old, stated willingness to comply with study procedures, were able to perform hand exercises, and had no known contraindications for 5% lidocaine cream. Additional exclusion criteria for participants included neurological impairments or conditions, pregnancy, and current use of illicit drugs or neuroactive medications. All study procedures were reviewed and approved by the University of Texas Rio Grande Valley Institutional Review Board, and participants provided informed consent.

Baseline characteristics assessment

Following enrollment and prior to TD application, subjects underwent baseline characteristic collection. We collected baseline characteristics to identify the potential influence of external factors on the amount of TD observed. Specifically, we evaluated sex, age, arm circumference, and body mass index. Participants verbally indicated their age and sex. Fat percentage and body mass index were measured using a handheld body fat analyzer (Omron HBF-306C, Omron Corporation, Kyoto, Japan). The circumference of the right biceps brachii was measured using a flexible centimeter tape around the thickest part of the muscle. We also evaluated baseline sensation of the right biceps using von Frey hair filament testing (VFHT) (outlined below).

Application of TD

We applied TD to the right biceps of all participants. In our study, we chose to focus on the upper extremity in a large muscle (biceps brachii), given the importance of reaching after many neurological conditions [32,33]. Further, previous work has suggested that excessive afferent input from this muscle may limit triceps recovery following neurological injury [34-36].

Prior to the application of TD, the participant self-cleaned their arm over the sink using soap and water. Next, to increase skin permeability, the skin of the right biceps area was exfoliated (NuPrep, Weaver and Company, Aurora, CO) and cleaned with alcohol pads. Following cleaning, 10 cubic centimeters (cc) or 10 grams of 5% lidocaine cream (Numb520, Ebanel, Brea, CA) was then applied across the area of their biceps. Lidocaine cream was measured prior to each session and applied using a sterile syringe. We spread the cream across the lateral, center, and medial biceps area. Since the amount of lidocaine cream exceeded the amount that could be absorbed by the skin, an approximate 1/8 to ¼ inch of cream remained on the surface of the skin. Therefore, to prevent the cream from transferring to clothing or other areas of the subject, following application, the entire area was covered using Tegaderm dressing.

Assessment of sensation with VFHT

We utilized VFHT to evaluate sensation before and after TD. VFHT is commonly used in the evaluation of the level of TD for its good validity and reliability in measuring the mechanical threshold of touch and determining tactile detection limits [15]. Further, given its mobility and ease of use, we sought to utilize it to aid in the translation of TD. For all VFHT testing (baseline and during TD), the participant was blindfolded. VFHT filaments were tested at nine distinct points, in a randomized order, on the participants’ biceps (Figure 1). Participants verbally indicated with a yes or no response if any sensation was felt. To account for bias, false negatives were also evaluated (e.g., no filament was placed, but the subject was asked if anything was felt) for all subjects. Sensation was measured every 15 minutes following TD application, assessing six total time points: baseline, 15, 30, 45, 60, and 75 minutes. For measurements during TD, the Tegaderm® dressing was removed while the cream remained in contact with the skin. VFHT filaments were cleaned between measurements.

We evaluated the sensation of the biceps brachii using 12 von Frey filaments at each timepoint. Filament sizes included the following: 2.36, 2.44, and 2.83 (normal); 3.22 and 3.83 (diminished light touch); 4.08, 4.17, and 4.31 (diminished protective sensation); 4.56, 4.74, 4.93, and 5.07 (loss of protective sensation).

Data analysis

We evaluated the sensitivity of the biceps for each filament at all evaluated timepoints. Percent sensitivity was defined as the number of positive verbal identifications of filament placement by the total number of testing times. Sensitivity profiles for each filament were generated by evaluating the percent sensitivity (y-axis) compared to time (x-axis).

For each participant, we averaged the percent sensitivity across all filaments within the respective four sensation categories at each timepoint (normal (2.36, 2.44, 2.83), diminished light touch (3.22, 3.83), diminished protective sensation (4.08, 4.17, 4.31), and loss of protective sensation (4.56, 4.74, 4.93, 5.07)). In addition, we also determined the mean sensitivity profile for each participant by averaging all percent sensitivity profiles at each timepoint. Thus, for each participant, we had four sensitivity profiles and a mean profile that were used for analysis.

Statistical analysis

Statistical analysis was completed using SPSS (v.28.0, 2021; IBM Corp., Armonk, NY). Differences in biceps sensitivity in the four sensation categories (normal sensation, diminished light sensation, diminished protective sensation, and loss of protective sensation) from baseline to 75 minutes were evaluated using a two-way repeated measures ANOVA (time x sensitivity category), followed by post hoc Bonferroni-corrected mean difference analyses. When the assumption of sphericity was violated, a Greenhouse-Geisser correction was applied.

We evaluated the relationship between patient characteristics and sensitivity changes using Pearson’s correlations. Stepwise multiple linear regression analyses were conducted to examine the effects of demographic variables on loss of protective sensation. Interaction terms between key demographics were included to assess whether these relationships varied across subgroups. Additionally, we performed a multiple linear regression to evaluate changes in VFHT filament-detected loss of protective sensation from baseline to 75 minutes. Post hoc t-tests were used to compare percent sensation at the 60-minute mark across VFHT filament groups. A p-value of 0.05 was considered statistically significant.

## Results

Subject characteristics

A total of 18 participants (males = 8, females = 10), aged 18 to 23 years (mean = 24.7 ± 11.3 (standard deviation (SD)), completed the study (Table 1). On average, participants had a healthy body mass index (BMI) (23.8 ± 5.3 (SD)), and the average arm circumference was 27.6 cm (± 4.6 cm (SD)). Due to data loss, demographic data were missing for two participants. We utilized linear regression prediction imputation to impute missing demographic data. Fat percentage was predicted in one participant using sex, age, BMI, and arm circumference as predictors (r = 0.91, p < 0.001). Arm circumference was predicted in one participant using sex, age, BMI, and fat percentage as predictors (r = 0.82, p < 0.001).

**Table 1:** Patient demographics. BMI: body mass index; cm: centimeters; SD: standard deviation.

Demographic	Mean (SD)/number (%)
Male	8 (44.4%)
Female	10 (55.6%)
Age	24.7 (11.3)
BMI	23.8 (5.3)
Fat %	22.3 (7.4)
Arm circumference (cm)	27.6 (4.6)

Changes in percent sensitivity profiles

**Figure 2:**
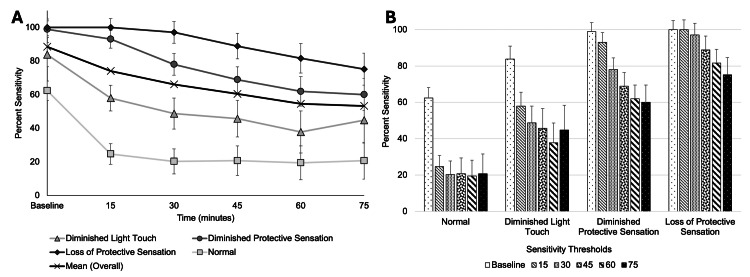
Sensation of the biceps following application of over-the-counter lidocaine cream from baseline to 75 minutes. (A) Percent sensitivity profiles for each sensory threshold of the von Frey hair test (VFHT), including overall mean. All sensory threshold levels showed a significant reduction in sensitivity by 75 minutes. (B) Amount of sensation for each sensory threshold shown as a function of time. Normal sensation was immediately reduced by 15 minutes after application of temporary deafferentation (TD). Loss of diminished light touch, diminished protective sensation, and loss of protective sensation were maximally achieved by 60 minutes after application.

We evaluated changes in biceps sensitivity over time for all VFHT filaments, expressed as percent sensitivity, for loss of normal sensation, diminished light touch, diminished protective sensation, and loss of protective sensation (Figure 2). Participants reported that they felt overall less biceps sensation over time (more TD) from baseline to 75 minutes, as indicated by a change in percent sensitivity thresholds (Figure 2A) (F(2.76, 632.98) = 83.99, p < 0.001, η² = 0.27). Additionally, there was an interaction of lower overall bicep sensation over time and the corresponding VFHT sensory evaluation thresholds (Figure 2B) (F(8.29, 632.98) = 6.81, p < 0.001, η² = 0.08). The interaction indicated that participants reported different degrees of sensation based on the filament thickness of the VFHT sensation thresholds being tested. We found that participants reported less bicep sensation (more TD) at 75 minutes compared to baseline through 45 minutes (p < 0.001). However, there were no statistically significant changes in bicep sensation between 60- and 75-minute time points (mean = -0.048, SE = 1.64, p > 0.05). This indicated that a duration of 60 minutes (minimum) was optimal for achieving loss of sensation.

Influence of baseline characteristics on the level of TD

Pearson correlations indicated that older participants (r = -0.31, p < 0.001) had lower total bicep sensitivity (more TD). No statistically significant relationships were found between sensitivity change for sex, fat percentage, BMI, and arm circumference (p > 0.05). As part of a sub-analysis, we evaluated the influence of baseline characteristics on loss of protective sensation threshold (filaments 4.56, 4.74, 4.93, and 5.07). We found that older age had a significant role in lower sensitivity levels (β = -0.34, p = 0.006), while sex, BMI, fat percentage, and arm circumference did not (p > 0.4). When accounting for demographic and anthropometric interactions, we found that males experienced a disproportionately greater increase in loss of protective sensation compared to females (β = -0.90, t = 2.58, p < 0.02) and that older age continued to have a significant role in lower sensitivity levels (β = -0.41, p < 0.001). The statistically significant interaction effect was only seen in loss of protective sensation (β = 0.57, t = 2.80, p < 0.01), indicating that a complex interplay of sex, age, BMI, fat percentage, and arm width influenced the degree of sensation loss.

Selecting a VFHT filament for TD

**Figure 3:**
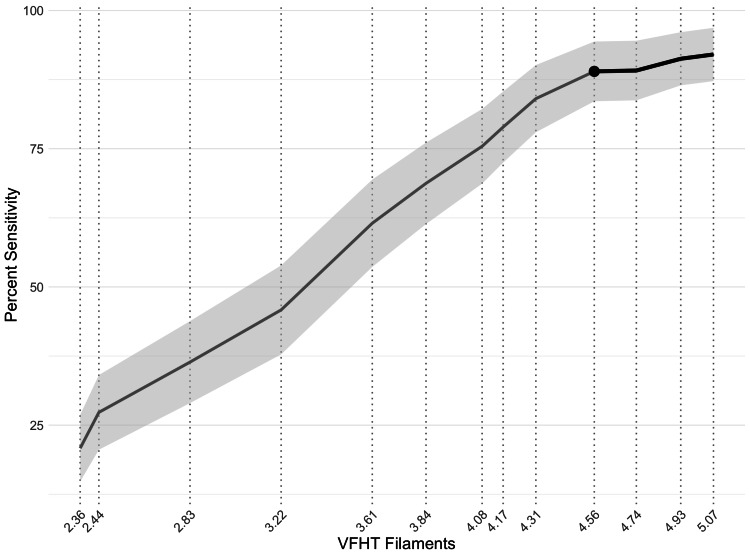
Identification of a von Frey hair filament to identify sensation loss with over-the-counter lidocaine cream for temporary deafferentation. We evaluated the percent sensitivity at 60 minutes for all von Frey hair filament test (VFHT) filaments tested. We observed that 4.56 was significant in reporting the participant’s level of biceps sensation from temporary deafferentation (TD) compared to other filaments in the “loss of protective sensation” threshold group.

To aid in the translation of TD to outpatient, community, and rural environments, we sought to determine if a single VFHT filament could accurately capture loss of bicep sensation. We focused our analysis at the timepoint of 60 minutes, since it was identified as the minimum time required for sensation loss. In addition, we focused our sub-analysis on filaments that assessed loss of protective sensation threshold since it was the least sensitive to change following TD application (Figure 2). According to multiple linear regression among the four VFHT filaments in protective sensation threshold (5.07, 4.93, 4.74, and 4.56), we found that only the 4.56 filament significantly aided in reporting the participants’ bicep TD from baseline to 75 minutes (β = -0.90, p = 0.019) (F(4, 103) = 5.21, p < 0.001, adj. R² = 0.14) (Figure 3).

When analyzing the 60-minute time point using ANOVA, we confirmed that TD could still be measured across the increasing thickness of VFHT filaments (F(12, 220) = 8.02, p < 0.001, η² = 0.30). Post hoc T-tests determined that 4.56 filament discriminated from 2.36 through 4.31 at 60 minutes (p < 0.05) (Table 2). Most importantly, the 4.56 filament did not detect TD any differently than the other filaments in the same sensory threshold group (protective sensation threshold) (p > 0.05). This evidence indicated that the 4.56 filament alone may identify loss of protective sensory threshold for TD.

**Table 2:** Percent sensation t-test comparisons between von Frey hair test (VFHT) filaments and the 4.56 filament at 60 minutes.

VFHT filaments	Sensory thresholds	Mean diff. (SE)	t	p
2.36	Normal	63.89 (9.26)	6.90	<0.001
2.44		62.96 (9.86)	6.39	<0.001
2.83		56.48 (10.01)	5.65	<0.001
3.22	Diminished light touch	47.22 (10.19)	4.63	<0.001
3.61		38.07 (9.57)	3.85	<0.001
3.84	Diminished protective sensation	30.25 (8.06)	3.75	0.001
4.08		18.52 (6.26)	2.96	0.002
4.17		16.98 (6.98)	2.43	0.009
4.31		8.64 (5.11)	1.69	0.026
4.74	Loss of protective sensation	0.00 (.00)	-	-
4.93		-0.31 (1.59)	-0.20	0.130
5.07		-4.01 (3.91)	-1.03	0.170

## Discussion

Overall, we found that TD could be safely and effectively achieved using OTC 5% lidocaine cream. Our results indicate that 60 minutes was the earliest time point to show a significant loss of protective sensation. Additionally, we found that sensitivity changes at 60 minutes could be reliably measured using VFHT filament 4.56, which could introduce an expeditious way of testing the desired sensory threshold sensitivity for TD. Under all the VFHT filaments in loss of protective sensation, 4.56 was also the only statistically significant predictor of percent sensation from baseline to 75 minutes. Our results suggest that using 5% lidocaine cream can achieve a similar effectiveness as studies that have used 5% eutectic mixture of local anesthetics EMLA for TD [5,11-13,28]. Specifically, EMLA has been known to be effective between 30 and 60 minutes for anesthesia measured with pinprick [37]. Here, our results have shown that 5% lidocaine cream needs 60 minutes or greater to have a loss of protective sensation (e.g., pinprick test), with heightened effectiveness on diminished protective sensation. Although it is important to note that while 5% lidocaine cream had a similar function for cutaneous anesthesia with a similar duration time, it had a slower onset of effectiveness, needing at least 60 minutes to reach the desired sensory threshold.

We found that age, sex, BMI, fat percentage, and arm circumference collectively influenced the loss of protective sensation threshold. While there is a complex relationship between demographic variables and TD, our analysis revealed that age and sex had statistically significant effects. Our findings build on previous studies suggest that as individuals get older, males may experience a steeper increase in TD compared to females [38]. Various physical means were implemented to enhance lidocaine permeation to ensure proper onset, such as skin exfoliation, degreasing with alcohol, and using Tegaderm to cover the targeted area [39]; however, the results witnessed may be due to different anatomical features between sexes that are influenced by age. Previous literature has suggested that there are sex differences in pressure pain threshold and pain sensitivity. For example, both healthy females and females with a stroke exhibit higher pain sensitivity compared to their male counterparts [40]. Additionally, other studies indicate that males possess more effective endogenous pain inhibitory systems and demonstrate greater activation of pain-regulation regions, such as the periaqueductal gray and amygdala [41]. Further, it has been indicated that such differences are potentially influenced by estrogen and progesterone, including aging, having an effect on the peripheral nervous system, leading to decreased tactile sensitivity and altered pain perception [42]. Specifically, research has linked sex hormones with sensory perception, where estrogen offered potential neuroprotective effects compared to testosterone [43]. In addition to the mechanisms of aging affecting the somatosensory system, aging leads to a reduction in spindle afferents, alterations in Golgi tendon organs, and a decline in Ruffini endings and Pacinian corpuscles. These factors contribute to a lower baseline sensory input in older individuals, potentially enabling them to achieve thermal discrimination more rapidly [44]. Taken collectively, it is likely that the steeper increase in TD we observed in our study in males may be attributed to both anatomical, hormonal, and age.

As part of our protocol, we sought to use an evaluation tool that could quickly, reliably, and effectively assess sensory changes and be used in rural and community settings. Based on their widespread use clinically, we chose to utilize VFHT in the present study. Currently, there is an absence of evidence in the literature on utilizing VFHT to evaluate the effectiveness of TD. However, other techniques that have been used to measure the efficacy of TD are not ideal for being self-administered at home, and may not be as user-friendly or scientifically reliable. For example, most studies employing TD have utilized MRI, sensory evoked potentials (SSEPs), or motor evoked potentials (MEPs) to confirm the effect of TD on improving hand sensory and fine motor functions [11]. Our data suggests that TD onset could be measured with one 4.56 VFHT filament, a method that could reduce the cost of implementation. For example, OTC topical lidocaine application could be applied by the patient, with a simple provided kit, without the need for in-person contact. Additionally, video conferencing could facilitate patient instruction to ensure protocol adherence.

Study limitations

Our study had several limitations that should be noted. First, since we were conducting a pilot study, a control group (e.g., lotion without lidocaine group) was not included. However, to enhance study integrity, we evaluated the incidence of false negatives during VFHT testing of sensitivity in all participants. For all participants, no false negatives were noted. Second, we acknowledge that the achievement of sensitivity was only assessed using VFHT, and no advanced sensory measurement system (e.g., SSEPs) was utilized. Thus, further data are required to compare how changes in VHFT may relate to changes in SSEPs or other measures of sensory loss. Finally, our study design resulted in no additional measurements of sensational loss beyond 75 minutes. We chose 75 minutes as an endpoint based on previous literature using topical creams for TD. It is possible that additional loss in protective sensation may be noted beyond 75 minutes, and further studies would need to be conducted to evaluate how additional time would influence sensation.

## Conclusions

TD is an innovative rehabilitative adjunct that has shown particular promise in healthy populations and populations with stroke. Current studies have utilized prescription-grade topical creams to achieve observed benefits. However, such approaches may limit widespread use and integration, particularly in clinical settings that do not have access to pharmacies or rural areas. Here, we sought to evaluate whether an OTC lidocaine cream would achieve the effects of TD. Overall, our data suggest that TD can be achieved using OTC lidocaine cream in a similar time frame to prescription-grade topical creams. Use of OTC lidocaine cream may be helpful in translating TD to rural and community-based populations or telemedicine. Future work should evaluate the effects of OTC topical cream on motor output and patient compliance in community settings.

## References

[1] Dick SH, Rasmusson DD (2002). Effects of temporary deafferentation on raccoon post-synaptic dorsal column neurons. Brain Res.

[2] Northgrave SA, Rasmusson DD (1996). The immediate effects of peripheral deafferentation on neurons of the cuneate nucleus in raccoons. Somatosens Mot Res.

[3] Rasmusson DD (1988). Projections of digit afferents to the cuneate nucleus in the raccoon before and after partial deafferentation. J Comp Neurol.

[4] Takeda M, Oshima K, Takahashi M, Matsumoto S (2009). Systemic administration of lidocaine suppresses the excitability of rat cervical dorsal horn neurons and tooth-pulp-evoked jaw-opening reflex. Eur J Pain.

[5] Björkman A, Rosén B, Lundborg G (2004). Acute improvement of hand sensibility after selective ipsilateral cutaneous forearm anaesthesia. Eur J Neurosci.

[6] Brasil-Neto JP, Valls-Solé J, Pascual-Leone A (1993). Rapid modulation of human cortical motor outputs following ischaemic nerve block. Brain.

[7] Werhahn KJ, Mortensen J, Kaelin-Lang A, Boroojerdi B, Cohen LG (2002). Cortical excitability changes induced by deafferentation of the contralateral hemisphere. Brain.

[8] Ziemann U, Corwell B, Cohen LG (1998). Modulation of plasticity in human motor cortex after forearm ischemic nerve block. J Neurosci.

[9] Murphy BA, Haavik Taylor H, Wilson SA, Knight JA, Mathers KM, Schug S (2003). Changes in median nerve somatosensory transmission and motor output following transient deafferentation of the radial nerve in humans. Clin Neurophysiol.

[10] Weiss T, Miltner WH, Liepert J, Meissner W, Taub E (2004). Rapid functional plasticity in the primary somatomotor cortex and perceptual changes after nerve block. Eur J Neurosci.

[11] Björkman A, Weibull A, Rosén B, Svensson J, Lundborg G (2009). Rapid cortical reorganisation and improved sensitivity of the hand following cutaneous anaesthesia of the forearm. Eur J Neurosci.

[12] Petoe MA, Jaque FA, Byblow WD, Stinear CM (2013). Cutaneous anesthesia of the forearm enhances sensorimotor function of the hand. J Neurophysiol.

[13] Rosén B, Björkman A, Lundborg G (2006). Improved sensory relearning after nerve repair induced by selective temporary anaesthesia - a new concept in hand rehabilitation. J Hand Surg Br.

[14] Sens E, Teschner U, Meissner W (2012). Effects of temporary functional deafferentation on the brain, sensation, and behavior of stroke patients. J Neurosci.

[15] Sehle A, Büsching I, Vogt E, Liepert J (2016). Temporary deafferentation evoked by cutaneous anesthesia: behavioral and electrophysiological findings in healthy subjects. J Neural Transm (Vienna).

[16] Björkman A, Rosén B, Lundborg G (2005). Enhanced function in nerve-injured hands after contralateral deafferentation. Neuroreport.

[17] Sadato N, Zeffiro TA, Campbell G, Konishi J, Shibasaki H, Hallett M (1995). Regional cerebral blood flow changes in motor cortical areas after transient anesthesia of the forearm. Ann Neurol.

[18] Sabbahi MA, De Luca CJ (1991). Topical anesthetic-induced improvements in the mobility of patients with muscular hypertonicity: preliminary results. J Electromyogr Kinesiol.

[19] Werhahn KJ, Mortensen J, Van Boven RW, Zeuner KE, Cohen LG (2002). Enhanced tactile spatial acuity and cortical processing during acute hand deafferentation. Nat Neurosci.

[20] Weiss T, Sens E, Teschner U, Meissner W, Preul C, Witte OW, Miltner WH (2011). Deafferentation of the affected arm: a method to improve rehabilitation?. Stroke.

[21] Muellbacher W, Richards C, Ziemann U (2002). Improving hand function in chronic stroke. Arch Neurol.

[22] Sens E, Knorr C, Preul C, Meissner W, Witte OW, Miltner WH, Weiss T (2013). Differences in somatosensory and motor improvement during temporary functional deafferentation in stroke patients and healthy subjects. Behav Brain Res.

[23] Sumitani M, Miyauchi S, Yozu A, Otake Y, Saitoh Y, Yamada Y (2010). Phantom limb pain in the primary motor cortex: topical review. J Anesth.

[24] Pușcașu C, Zanfirescu A, Negreș S (2023). Recent progress in gels for neuropathic pain. Gels.

[25] Cohen SP, Caterina MJ, Yang SY, Socolovsky M, Sommer C (2024). Pain in the context of sensory deafferentation. Anesthesiology.

[26] Sturma A, Hruby L, Vujaklija I, Østlie K, Farina D (2021). Treatment strategies for phantom limb pain. Bionic Limb Reconstruction.

[27] Sultana A, Singla RK, He X, Sun Y, Alam MS, Shen B (2021). Topical capsaicin for the treatment of neuropathic pain. Curr Drug Metab.

[28] Rosén B, Björkman A, Lundborg G (2008). Improved hand function in a dental hygienist with neuropathy induced by vibration and compression: the effect of cutaneous anaesthetic treatment of the forearm. Scand J Plast Reconstr Surg Hand Surg.

[29] Choukou MA, He E, Moslenko K (2023). Feasibility of a virtual-reality-enabled at-home telerehabilitation program for stroke survivors: a case study. J Pers Med.

[30] Bustamante-Vázquez JL, Rodrigo-Morales GJ, De-Dios-Pérez JI, Artiles-Sánchez J, Barragán Carballar C, Alonso-Pérez JL, Villafañe JH (2024). Optimizing telehealth strategies for rehabilitation: recommendations from rural physical therapists. Top Geriatr Rehabil.

[31] Opsommer E, Zwissig C, Korogod N, Weiss T (2016). Effectiveness of temporary deafferentation of the arm on somatosensory and motor functions following stroke: a systematic review. JBI Database System Rev Implement Rep.

[32] de Bruin M, Veeger HE, Kreulen M, Smeulders MJ, Bus SA (2013). Biceps brachii can add to performance of tasks requiring supination in cerebral palsy patients. J Electromyogr Kinesiol.

[33] Barker RN, Brauer S, Carson R (2009). Training-induced changes in the pattern of triceps to biceps activation during reaching tasks after chronic and severe stroke. Exp Brain Res.

[34] Potter-Baker KA, Janini DP, Frost FS (2016). Reliability of TMS metrics in patients with chronic incomplete spinal cord injury. Spinal Cord.

[35] Potter-Baker KA, Janini DP, Frost F, Varnerin N, Cunningham DA, Sankarasubramanian V, Plow EB (2016). A picture is worth a 1000 words: the potential of defining incompleteness of injury with neuroimaging and brain neurophysiology. J Spinal Cord Med.

[36] Potter-Baker KA, Lin YL, Plow EB (2017). Understanding cortical topographical changes in liminally contractable muscles in SCI: importance of all mechanisms of neural dysfunction. Spinal Cord.

[37] Gajraj NM, Pennant JH, Watcha MF (1994). Eutectic mixture of local anesthetics (EMLA) cream. Anesth Analg.

[38] Sens E, Franz M, Preul C, Meissner W, Witte OW, Miltner WH, Weiss T (2018). Effects of temporary functional deafferentation in chronic stroke patients: who profits more?. Neural Plast.

[39] Kumar M, Chawla R, Goyal M (2015). Topical anesthesia. J Anaesthesiol Clin Pharmacol.

[40] Zhang YH, Wang YC, Hu GW (2021). The effects of gender, functional condition, and ADL on pressure pain threshold in stroke patients. Front Neurosci.

[41] Chesterton LS, Barlas P, Foster NE, Baxter DG, Wright CC (2003). Gender differences in pressure pain threshold in healthy humans. Pain.

[42] Eltumi HG, Tashani OA (2017). Effect of age, sex and gender on pain sensitivity: a narrative review. Open Pain J.

[43] Herrera-Rangel A, Aranda-Moreno C, Mantilla-Ochoa T, Zainos-Saucedo L, Jáuregui-Renaud K (2014). The influence of peripheral neuropathy, gender, and obesity on the postural stability of patients with type 2 diabetes mellitus. J Diabetes Res.

[44] Shaffer SW, Harrison AL (2007). Aging of the somatosensory system: a translational perspective. Phys Ther.

